# Musashi-1 Post-Transcriptionally Enhances Phosphotyrosine-Binding Domain-Containing m-Numb Protein Expression in Regenerating Gastric Mucosa

**DOI:** 10.1371/journal.pone.0053540

**Published:** 2013-01-04

**Authors:** Tetsufumi Takahashi, Hidekazu Suzuki, Takao Imai, Shinsuke Shibata, Yoshiaki Tabuchi, Kanji Tsuchimoto, Hideyuki Okano, Toshifumi Hibi

**Affiliations:** 1 Division of Gastroenterology and Hepatology, Department of Internal Medicine, School of Medicine, Keio University, Tokyo, Japan; 2 Laboratory of Pathophysiology, Division of Clinical Medicine, School of Pharmacy, Kitasato University, Tokyo, Japan; 3 Department of Physiology, School of Medicine, Keio University, Tokyo, Japan; 4 Division of Molecular Genetics, Life Science Research Center, University of Toyama, Toyama, Japan; Massachusetts General Hospital, United States of America

## Abstract

**Objective:**

Upregulation of the RNA-binding protein Musashi-1 (Msi1) has been shown to occur in rat gastric corpus mucosa after ethanol-induced mucosal injury. However, there is no direct evidence linking Msi1 with gastric regeneration. We examined the process of tissue repair after acute gastric mucosal injury with Msi1-knock-out (KO) mice to clarify the role of Msi1 and Msi1-dependent regulation of m-Numb expression in regenerating gastric mucosa.

**Methods:**

Acute gastric injury was induced in Msi1-KO and wild-type ICR mice by administering absolute ethanol. Expression of the splicing variants of *m-Numb* mRNA and protein in the gastric mucosa were analyzed by quantitative RT-PCR and western blotting, respectively.

**Results:**

We demonstrated that phosphotyrosine-binding domain-containing m-Numb expression was significantly upregulated at both the mRNA and protein levels in wild-type mice at 3 h after ethanol-induced acute gastric injury. In contrast, in Msi1-KO mice, the m-Numb protein was expressed weakly, and was associated with delayed regeneration of the injured gastric mucosal epithelium. In the Msi1-KO mouse, the ratio of *m-Numb* mRNA to total *m-Numb* mRNA in the heavy polysome fractions was lower than that in the wild-type mouse. Further, we showed that m-Numb-enhancement in gastric mucous cells induced the expression of prostate stem cell antigen and metallothionein-2. Under the m-Numb enhancing condition, the gastric cells exhibited enhanced cell proliferation and were significantly more resistant to H_2_O_2_-induced cell death than control cells.

**Conclusions:**

Msi1-dependent post-transcriptional enhancement of m-Numb is crucial in gastric epithelial regeneration.

## Introduction

Epithelia in the digestive tract exhibit a remarkable capacity for regeneration. The gastric mucosa, in particular, shows continuous regenerative activity, mediated through the differentiation of stem cells, which may be localized in a proliferating area called the isthmus, and migration of the differentiating cells towards the gastric surface and into the base of the fundic glands. Gastric mucosal architecture is restored within a very short period following injury [1], [2], [3]. However, the mechanisms underlying this rapid restoration of the gastric mucosal architecture have not yet been fully clarified.

An RNA-binding protein, Musashi-1 (Msi1) [4], [5], [6] was isolated as a mammalian homologue of the *Drosophila* Musashi; this protein is required for the asymmetric cell division of the sensory neural precursor cells [7], [8]. In the mammalian central nervous system, Msi1 is known to regulate progenitor cell function through the post-transcriptional regulation of its target RNA [4], [5], [6]. In the rat gastric corpus, Msi1 expression has been shown to be upregulated after ethanol-induced mucosal injury [9]. However, currently, there is no direct evidence linking Msi1 with gastric regeneration.

Msi1 is known to bind to the 3′-untranslated region (UTR) of several target mRNAs and to regulate these genes post-transcriptionally [10], [11], [12], [13], in particular, those encoding Numb and p21. Msi1-dependent suppression of Numb translation has been reported to contribute to the self-renewal of neural stem cells [11]. Recent studies indicated that deregulation of the Musashi/Numb pathway is likely to be involved in tumor development [14], [15], [16]. Numb expression has been demonstrated in the gastric epithelium during the early stages of chicken gastric gland formation [17], although its expression and translational regulation by Msi1 in the postnatal gastric mucosa remains unclear.

Numb was first identified in *Drosophila* as a protein playing a role during asymmetric cell division of the neural precursors [18], [19]. The mammalian homolog of *Drosophila* Numb (m-Numb) and Numb-like (a second mammalian Numb protein) [20] have been shown to be key factors involved in the regeneration of the brain after ventricular damage, since severe damage was observed in mice with conditional deletion of these genes in the postnatal brain [21]. The corresponding *m-Numb* gene has splicing sites affecting the phosphotyrosine-binding (PTB) domain and proline-rich region (PRR), and gives rise to at least 4 alternatively spliced transcripts [22], which produce 4 protein isoforms: Numb1 (72 kDa), Numb2 (66 kDa), Numb3 (71 kDa), and Numb4 (65 kDa) (Figure S1). These m-Numb splicing variants are not considered components of a single protein but rather as a family of stage-specific proteins with distinct regulatory roles in neural development [23], [24]. Furthermore, the expression of the individual splicing variants of *m-Numb mRNA* have been reported to differ in the adult testis, liver, lung, spleen, thymus, and brain [24], [25]. This indicates that splicing variant-specific m-Numb expression may be regulated in both temporal and a tissue-specific manner. The postnatal roles of each m-Numb variant in the gastric mucosa remain unclear.

In the present study, we demonstrate that splicing variant-specific m-Numb expression is induced during gastric mucosal regeneration after acute damage, and that the stomachs of Msi1-knock-out (Msi1-KO) mice, which lack the m-Numb expression response, show delayed gastric regeneration. The m-Numb protein induces expression of regeneration-related genes such as prostate stem cell antigen (PSCA) and metallothionein-2 (Mt2). We report that this enhancement of m-Numb protein expression by Msi1 in the stomach mucosal regeneration is occurred by post-transcriptional regulation.

## Results

### Delayed Gastric Mucosal Repair after Ethanol Administration in Msi1-KO Mice

**Figure 1:**
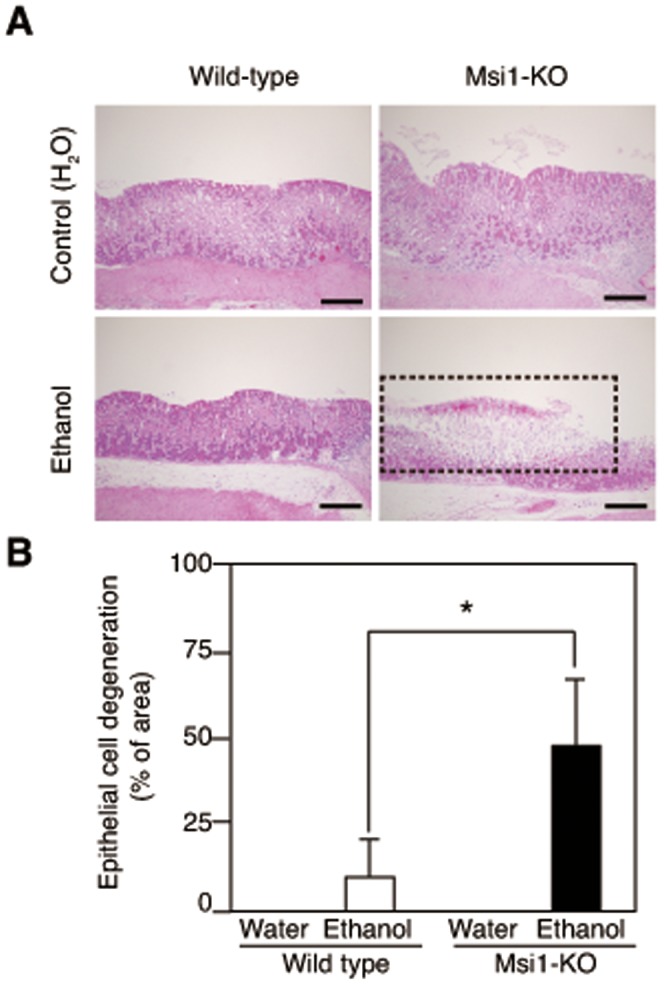
Gastric epithelial cell degeneration after gastric damage. (A) Hematoxylin and eosin (H&E)-stained sections of the stomachs of wild-type and Msi1-KO mice. Control group (water administration) and ethanol administration group. Bars = 100 µm (B) Gastric epithelial cell degeneration in the fundic area was noted in the H&E-stained sections. *P<0.05.

**Figure 2:**
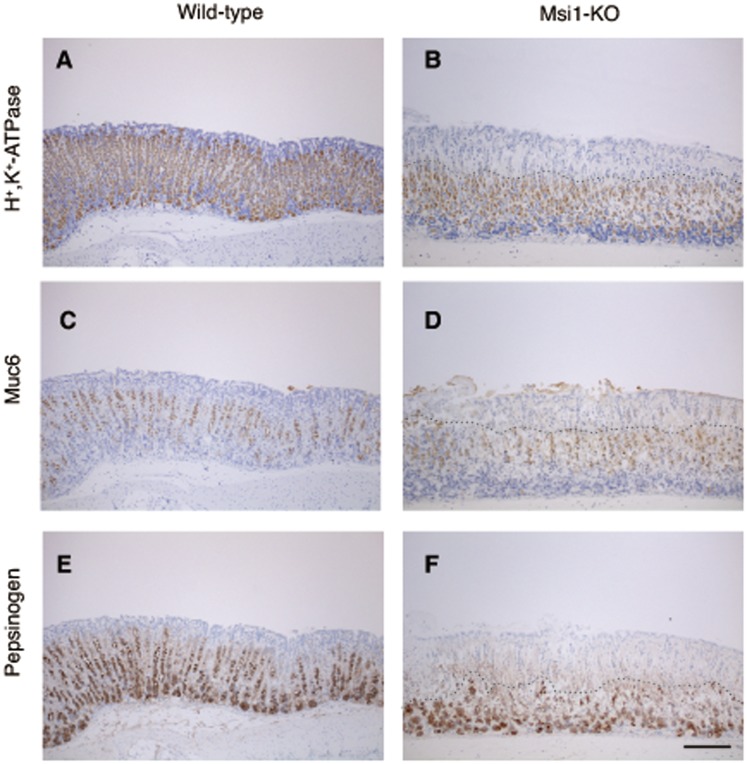
Immunohistochemical analysis revealed a defect in cell differentiation in Msi1-KO stomach. Wild-type (A, C, and E) and Msi1-KO (B, D, and F) mice were administered ethanol. Sections of the gastric mucosa from each mouse at 5 h after ethanol administration were then stained using anti-H^+^, K^+^-ATPase- (A and B), anti-Muc6- (C and D), and anti-pepsinogen- (E and F) antibodies. Bar = 100 µm.

Msi1 is up-regulated in rat gastric corpus mucosa after ethanol-induced mucosal injury [9]. We firstly examined the histochemical analysis of gastric mucosa of wild-type and Msi1-KO mice in the water-treated control group. There was no significant difference in the erosive lesions of gastric mucosa (Figure 1A) and the number of H^+^, K^+^-ATPase-positive parietal cells, Muc6-positive mucous neck cells, and pepsinogen-positive mucosal zymogenic cells between wild-type (n = 5) and Msi1-KO (n = 3) mice in the control group (Figure S2). Thus, we investigated mucosal regeneration after ethanol-induced acute gastric injury in Msi1-KO mice. Five hours after ethanol administration, the erosive and ulcerative lesions in the gastric mucosa were more enhanced in the Msi1-KO mice than in the wild-type mice (Figure 1A). The area of erosive and ulcerative lesions was significantly larger in the stomach of ethanol-treated Msi1-KO mice (n = 3) than in the stomach of ethanol-treated wild-type mice (n = 6) (Figure 1B). Furthermore, at this time-point, an abundant decrease in the number of H^+^, K^+^-ATPase-positive parietal cells, Muc6-positive mucous neck cells, and pepsinogen-positive mucosal zymogenic cells was observed in the superficial area of the gastric fundus of the Msi1-KO mice (above on dotted line in Figures 2B, D, and F), even in areas of undetached epithelium.

### Post-transcriptional Enhancement of m-Numb Expression by Msi1 in the Stomach

**Figure 3:**
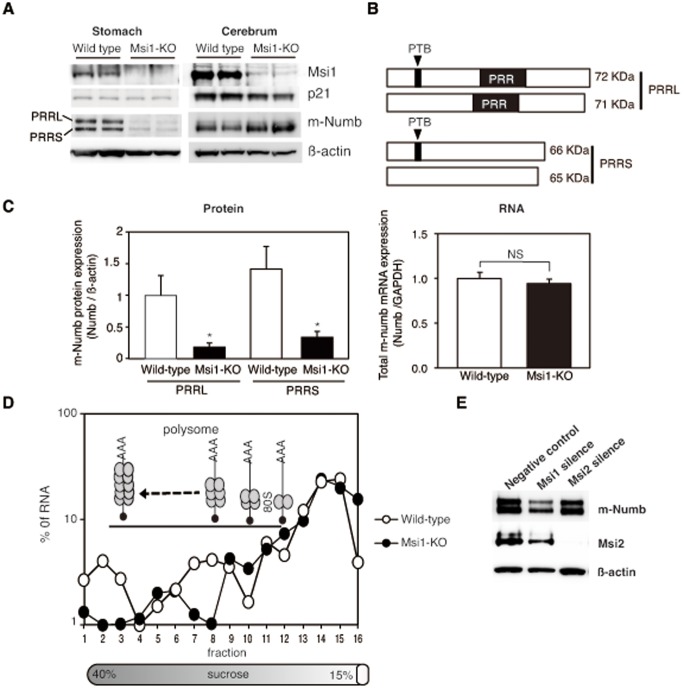
Expression of p21 and m-Numb in the stomach of wild-type and Msi1-KO mice. (A) Western blots indicating expression of Msi1, p21, and m-Numb protein in the stomach and cerebrum of sham-treated wild-type and Msi1-KO mice. (B) Classification of m-Numb protein by proline-rich region (PRR). (C) m-Numb protein and total RNA expression. Western blotting and quantitative RT-PCR for the expression analysis of m-Numb protein and mRNA in sham-treated wild-type and Msi1-KO mice was performed in triplicate. The density of each m-Numb protein band in western blotting is normalized to actin and represented as the fold-change relative to expression of the protein in wild-type mice. White bars: wild-type mice; black bars: Msi1-KO mice. *P<0.01 compared to wild-type mice. (D) Polysome analysis of *m-Numb* mRNA from mouse stomach. Fractions of a polysome gradient prepared from the stomachs of wild-type (empty circles) and Msi1-KO (filled circles) mice. RNA was extracted from each fraction and used for quantitative RT-PCR. The results are shown as the percentage of the total amount of RNA in each fraction. (E) Knockdown of human *Msi1* and *Msi2* in N87 cells by shRNA-containing lentiviral particles. Western blotting was performed using primary antibodies specific for Msi1, Msi2, m-Numb, and β-actin.

To investigate whether the *Msi1* target gene was deregulated in the gastric mucosa of Msi1-KO mice, the expressions of p21 and m-Numb proteins, known to be the major targets of Msi1 in the stomach and cerebrum, were compared between wild-type and Msi1-KO mice. There was no difference in the expression of p21 protein in both the stomach and the cerebrum of Msi1-KO mice compared to that in the wild-type mice (Figure 3A). On the other hand, although the expression of m-Numb protein in the cerebrum was higher in Msi1-KO mice than in the wild-type mice, the expression of m-Numb protein in the stomach was markedly downregulated in the Msi1-KO mice (Figure 3A). The m-Numb protein was normally expressed in the gastric epithelium and slightly expressed in the zymogenic region (Methods S1 and Figure S3). And the decreased m-Numb expression in the Msi1-KO mice was not observed in other tissue like cerebrum, cerebellum, colon, testis, liver and lung (Figure S4).

The antibody used here for western blotting analysis can distinguish between the PRRL (Numb1 and/or Numb3) and PRRS (Numb2 and/or Numb4) forms of the m-Numb protein (Figure 3B), but not the PTB form, because of its small molecular size (11-amino acids) compared to PRR (49-amino acids); thus, western blotting with this antibody would yield 2 bands according to the presence or absence of PRR. In the stomach, the expression of PRRL and PRRS m-Numb proteins were significantly decreased in Msi1-KO mice as compared to that in wild-type mice (Figure 3C, n = 3, respectively).

The total *m-Numb* mRNA detected with the common primers for all the *m-Numb* variants was not decreased in the stomach of Msi1-KO mice as compared with that of wild-type mice, indicating post-transcriptional regulation of m-Numb protein expression by Msi1. Since the translated RNA forms a functional translational initiation complex, which consists of matured RNA and numerous 80S ribosomes, the polysome profiles in 15–40% sucrose gradients were established for lysates of wild-type and Msi1-KO mouse stomachs, and the pattern of *m-Numb* mRNA was determined by quantitative RT-PCR. The ratio of *m-Numb* mRNA in the heavy polysome fraction (fractions 1–9) was decreased in Msi1-KO mice (Figure 3D), indicating that translation of *m-Numb* mRNA was reduced in the stomach of Msi1-KO mouse.

The mammalian Musashi family consists of 2 genes, *Msi1* and *Msi2*. To investigate a possible compensatory effect of *Msi2* on the regulation of m-Numb in the stomach of Msi1-KO mice, *in vitro* knockdown analysis was performed in human gastric cell line N87 by using shRNA-containing lentivirus. Silencing *Msi1* reduced the expression of m-Numb in accordance with the results observed in the stomachs of Msi1-KO mice (Figure 3E). The Msi2 expression level was slightly decreased by *Msi1* silencing; however, silencing *Msi2* did not alter the expression of m-Numb, indicating that the enhanced expression of m-Numb protein is independent of *Msi2*.

### Enhanced m-Numb Protein Expression in the Regenerating Mouse Gastric Tissue

**Figure 4:**
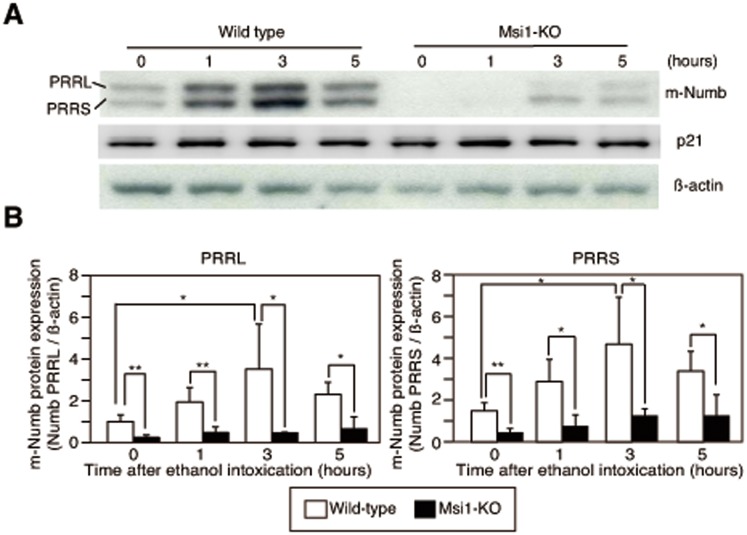
Expression of m-Numb protein in the stomach of wild-type and Msi1-KO mice after ethanol-induced gastric damage. (A) Expression of m-Numb and p21 protein in the stomach of wild-type and Msi1-KO mice. (B) The intensity of each band in the western blot of m-Numb was analyzed and the results were statistically compared. White bars: wild-type; black bars: Msi1-KO. *P<0.05, **P<0.01.

To investigate changes in the expression of m-Numb protein after gastric injury, total protein was extracted from the gastric tissues of wild-type and Msi1-KO mice at 1, 3, and 5 h after ethanol administration, and these extracts were used in western blotting. The expression of both the PRRL and PRRS forms of m-Numb increased in a time-dependent manner after the induction of gastric damage in the wild-type mice (Figures 4A and B). In contrast, in the Msi1-KO mice, only a weak and insignificant induction of m-Numb expression was observed, and the m-Numb expression levels were significantly lower than those in wild-type mice at 1, 3, and 5 h after ethanol administration (Figures 4A and B). The expression of p21 did not change after the gastric damage (Figure 4A).

### Association of PTB-containing Splicing Variants of m-Numb mRNA with Mucosal Injury

**Figure 5:**
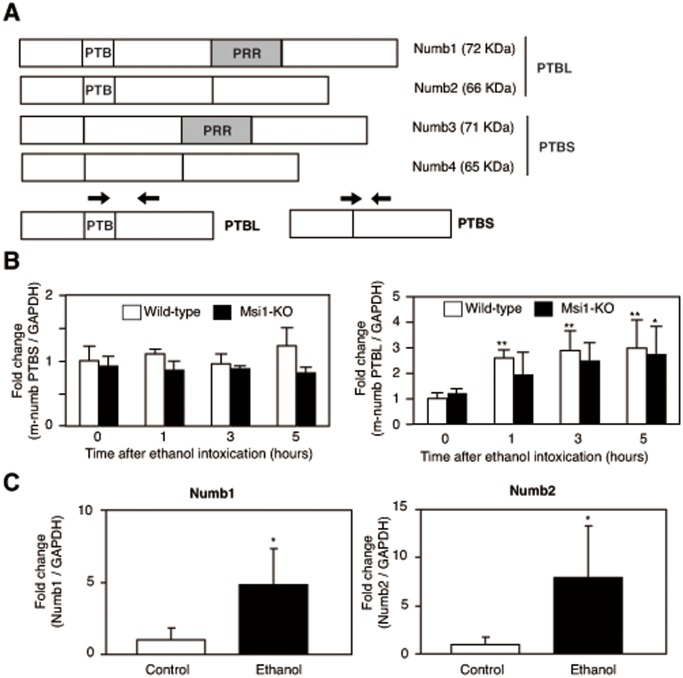
Expression levels of the m-Numb splicing variants in the stomach. (A) Schematic representation of the *m-Numb* gene and the primer designed for m-Numb *mRNA* amplification. (B) Expression of the PTBS- and PTBL-types of *m-Numb mRNA* in the stomach of ethanol-administered wild-type (white bars) and Msi1-KO (black bars) mice. Absolute ethanol was administered to both groups of mice and the mRNA expression of each of the splicing variants of *m-Numb* was analyzed by real-time PCR using SYBR Green. The expression levels were expressed as fold-change relative to the expression in the wild-type animals at 0 h. GAPDH was used as internal standard. (C) Semi-quantitative PCR was performed to confirm the expression of the complete PTBL-type of *m-Numb mRNA*, which was induced after gastric damage (at 5 h after ethanol administration). The primers used were the PTBL forward primer, and PRRS and PRRL reverse primers. *GAPDH* was used as the internal standard. The intensity of each band was analyzed, and the results are shown as fold-change relative to the expression in wild-type mice. *P<0.05 compared to Msi1-KO mice at 0 h, **P<0.01 compared to wild-type mice at 0 h.

Although m-Numb protein expression was confirmed after gastric injury, western blotting analysis could not distinguish the presence or absence of the PTB domain of the Numb protein. To distinguish the *m-Numb* splicing variants responsible for mucosal regeneration, total RNA was extracted from the gastric tissues of the wild-type (n = 5 each) and Msi1-KO mice (n = 3 each) at 1, 3, and 5 h after ethanol administration, and quantitative RT-PCR was performed using primers specific for each splicing variant (Figure 5A). The mRNA expression of the PTBS form of *m-Numb* did not change after ethanol-induced gastric damage (Figure 5B). The possibility of genomic contamination in the PCR results for PTBS was ruled out by reverse transcription in the absence of the sample (Figure S5). In contrast, the expression of the PTBL form of *m-Numb* mRNA was specifically increased after ethanol-induced gastric mucosal damage in both wild-type and Msi1-KO mice (Figure 5B).

Two forms of PTB-site-conserved complete *m-Numb* mRNA have been described, namely, Numb1 (PTBL-PRRL) and Numb2 (PTBL-PRRS), but the expression of each of these mRNAs could not be analyzed by quantitative PCR, because the PTB domain is distant from the PRR domain. Therefore, semi-quantitative PCR with a long-range DNA polymerase was performed for wild-type control (n = 5) and ethanol-administered (n = 5) mice. This indicated that the expression levels of both PTB-domain-containing *m-Numb* mRNAs were significantly increased at 5 h after ethanol administration (Figure 5C). Interestingly, as we demonstrated in Figure 4, the protein translation of the increased transcripts in ethanol administration was required for Msi1 activity.

### m-Numb Induced the Regeneration-related Gene Expression

**Figure 6:**
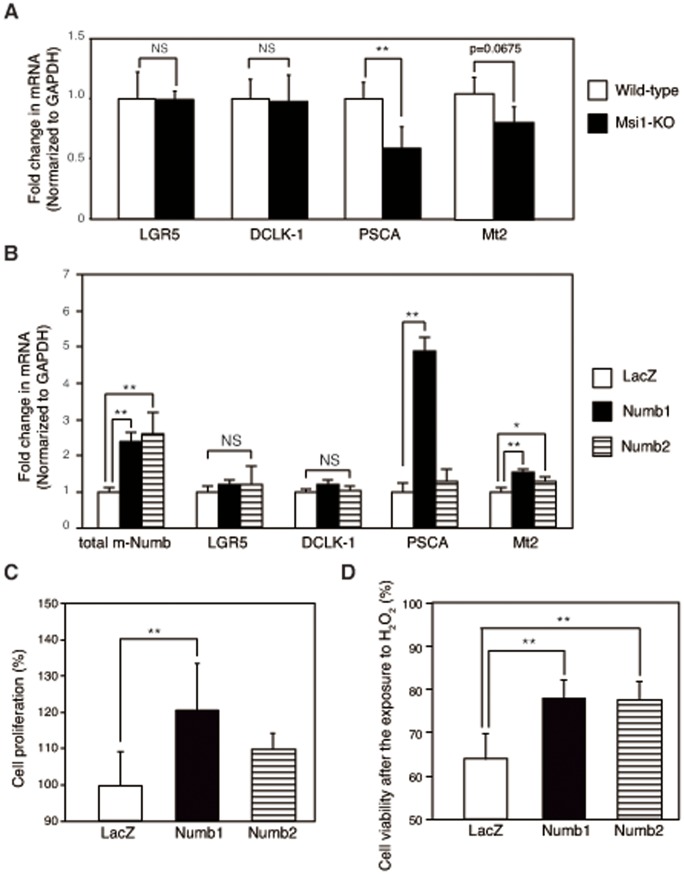
m-Numb-induced expression of regeneration-related genes. (A) Expression of *LGR5*, *DCLK1*, *PSCA*, and *Mt2* mRNA in the stomachs of sham-treated wild-type (white bars) and Msi1-KO (black bars) mice. **P<0.01 compared to wild-type mice. (B) mRNA expression of total *m-numb*, *LGR5*, *DCLK1*, *PSCA*, and *Mt2* in LacZ-, Numb1-, and Numb2-overexpressing MGE507 cells. *P<0.05, **P<0.01 compared to LacZ-overexpressing cells. (C) Cell proliferation assay in LacZ-, Numb1-, and Numb2-overexpressing MGE507 cells. **P<0.01 compared to LacZ-overexpressing cells. (D) H_2_O_2_-induced changes in cell viability in LacZ-, Numb1-, and Numb2-overexpressing MGE507 cells. **P<0.01 compared to LacZ-overexpressing cells.

To investigate the role of the induced m-Numb protein in regenerating gastric mucosa, changes in gastric regeneration-related genes were examined in the gastric mucosa of wild-type (n = 5) and Msi1-KO mice (n = 3). The mRNA expression of *leucine-rich repeat-containing G protein-coupled receptor 5* (*LGR5*) and *doublecortin and Ca^2+^/calmodulin-dependent kinase-like-1* (*DCLK1*), which are putative stem cell markers, did not change in either group of mice (Figure 6A). On the other hand, the mRNA expression of PSCA, which is expressed in the isthmus of gastric mucosa [26], was significantly decreased in Msi1-KO mice as compared to that in wild-type mice. In addition, antioxidant factor Mt2 expression tended to be lower in Msi1-KO mice than that in the wild-type mice. The expression of PSCA and Mt2 mRNA expression was significantly induced by the overexpression of Numb1 in mouse gastric cell line, MGE507 cells (Figure 6B) as compared to that in the LacZ control (n = 4 each). Mt2 expression was also induced by Numb2 overexpression (Figure 6B). In such a gastric cells, a significant increase of the proliferative activity in PRR containing Numb1-enhanced cells was observed as compared to that in LacZ control cells (*P*<0.001; Figure 6C). In contrast, no induction of the cell proliferation activity was observed following PRR not-containing Numb2 enhancing under the same conditions (Figure 6C). Additionally, H_2_O_2_-induced cell death was significantly inhibited in Numb1 and Numb2 enhancing cells compared to that in LacZ control cells (Figure 6D).

## Discussion

The present study is the first to demonstrate that upregulation of m-Numb protein expression in gastric tissues after acute gastric mucosal injury is mediated, at least in part, by Msi1. Since splicing variants of the m-Numb protein are considered to be a family of stage-specific proteins with distinct regulatory roles in neural development [23], we considered it important to clarify the expression of each splicing variant of m-Numb in this study. However, the antibody used in the study recognized the C-terminal region of the m-Numb protein, so that western blotting analysis could distinguish the presence of PRR among the m-Numb variants, but not that of PTB, because of the similarity in molecular weights. For this purpose, quantitative RT-PCR was performed using specific primers for each *m-Numb* splicing variant; this revealed splicing variant-specific induction of the PTB domain-containing *m-Numb* mRNA following gastric damage, indicating that the expression of Numb1 and Numb2, which contain PTB, is induced by gastric mucosal damage. On the other hand, although *m-Numb* mRNA was transcribed at the same level in Msi1-KO mice as in wild-type mice following gastric damage, m-Numb protein expression was decreased in the former. This suggested that m-Numb protein expression after gastric damage was post-transcriptionally downregulated by Msi1 ablation. Polysome analysis confirmed this post-transcriptional *m-Numb* regulation by Msi1.

The post-transcriptional downregulation of m-Numb protein expression by canonical Msi1-Numb-Notch axis regulation [16] has been found in wild type cerebral tissue (Figure 3A), glioblastoma U251MG [27], leukemia cells [14], [15] and fibroblast NIH3T3 cells [11]. Therefore, we expected that m-Numb protein expression would be enhanced in Msi1-KO mice, as compared to wild-type mice. However, the expression of m-Numb protein in the stomach of these mice was unexpectedly downregulated compared to that in wild-type mice.

Now, we could not solve the problem about whether this posttranscriptional enhancement in the stomach was exceptional. Notably, a recent report indicates that Msi1 induces the translation of *Robo3* mRNA by 3′-UTR–independent regulation in a particular neuron in the cerebellum [28]. The stomach-specific enhancement of m-Numb translation by Msi1 may be due to the stomach-specific expression of other RNA-binding protein(s), which are involved in mRNA stabilization/translation or by a putative translational repressor of m-Numb. The molecular mechanism underlying stomach-specific regulation of *m-Numb* translation remains to be elucidated.

In several variants of m-Numb, the PRRS type of m-Numb decreases the amount of nuclear Notch than the PRRL type of m-Numb, and it inhibits cell proliferation and promotes differentiation [29]. This regulation system is identified as the canonical Numb-Notch axis. On the other hand, the PRRL type of Numb promotes cell proliferation, possibly through the noncanonical Numb-Notch axis [22]. In addition, it is reported that the PTBL type of m-Numb does not induce Notch activation [30]. In this study, we showed the decreased expression of the PTBL-PRRL (Numb1) and PTBL-PRRS (Numb2) types of m-Numb in the gastric mucosa of Msi1-KO mice with delayed regeneration, indicating that the canonical Msi1-Numb-Notch axis is not suitable for gastric tissue.

Msi1 is reported to bind to the 3′-UTR of targeted RNA, and it binds to polyA-binding protein (PABP) [31]. The interaction between Msi1 and PABP inhibits initiation of translation by competing with translation-initiation factor eIF4G for PABP [31]. Therefore, the tissue-specific variation of the *m-Numb* UTR sequence may be important for this regulation. In order to identify specific features in *m-Numb* mRNA in stomach tissues, we analyzed the structures of *m-Numb* mRNA expressed in the stomach (Methods S1). 3′-RACE yielded only the full-length 3′-UTR sequence of the *m-Numb* mRNA, which corresponds to the reference sequence (positions 2276–3644 in accession NM_001005743.1) and contains Msi1 binding site. On the other hand, 5′-RACE analysis revealed the existence of gastric-specific ΔEx2-type variants, which lack exon 2, in the 5′-UTR sequence of the *m-Numb* mRNA (Figure S6 and Result S1). Thus, it is possible that the competing reaction is attenuated in mRNAs with a ΔEx2-type 5′-UTR via conformational change, resulting in translational activation of *m-Numb*.

The present model of acute gastric injury showed that the expression of the PTB domain-containing m-Numb protein was upregulated in the damaged gastric tissues of wild-type mice, especially at the site of origin of regeneration. Furthermore, Msi1-KO mice showed weak expression of m-Numb and delayed gastric regeneration after the mucosal injury. Since the expression of the other major target gene of Msi1, i.e., *p21*, showed no change following acute gastric damage, inducible m-Numb expression may be a key event in the regulation of gastric regeneration by Msi1. Numb1, one of the two m-Numb variants upregulated after gastric damage, has the ability to induce cell proliferation, which is important for tissue regeneration. Although PRR of Numb1 functions as SH3-binding domain and induces cell proliferation [22], the proliferation mechanism has been unclear. In this connection, we demonstrated the induction of PSCA expression and cell proliferation by Numb1enhancement in gastric cells. PSCA promotes cell proliferation through the regulation of cell cycle in prostate cell [32], suggesting that Numb1 may promote gastric cell proliferation by PSCA induction. The PSCA induction may be due to the interaction between Numb1 and transcriptional factor, which has SH3 domain. Furthermore, the PSCA is also involved in cell renewal [33] and is expressed in the isthmus of human gastric mucosa, which may contain differentiating progenitor cells [26]. The C allele of rs2294008 in *PSCA*, which results in altered subcellular localization and stability of the protein, is reported to be associated with increased risk of ulceration in metaplasia consisting of gastric-type mucous secreting cells [34]. Thus, Numb1 may promote gastric regeneration through the regulation of progenitor cell function like cell proliferation by the induction of PSCA.

**Figure 7:**
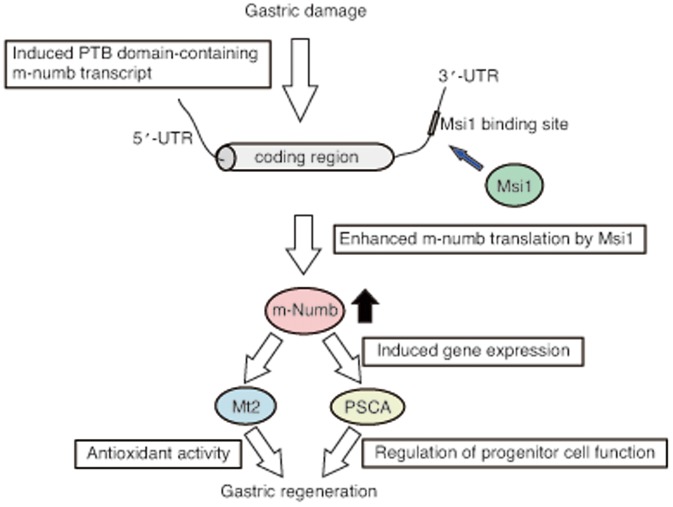
Schematic representation of Msi1-dependent gastric regeneration. After gastric damage, PTB domain-containing *m-numb* transcript is induced. Msi1 enhances the *m-numb* translation. The translated m-Numb protein induces the expression of regeneration-related genes such as *PSCA* and *Mt2*, resulting in gastric regeneration.

In addition, PTBL type of m-Numb, Numb1 and Numb2, induced mRNA expression of *Mt2*, which has anti-oxidant activity. Metallothionein transcription is mainly regulated by metal-regulatory transcription factor 1 (MTF-1), which binds to DNA sequence motifs of metallothionein, known as metal response elements (MREs) via the zinc finger domain of MTF-1 [35], [36], [37]. So induced Mt2 mRNA expression by PTBL type of m-Numb may be due to the regulation of MTF-1-MREs binding activity or the direct binding of ligand of Numb, LNX, which interacts to Numb PTB [38], [39] and contains zinc fingers. Mt2-induced gastric cells by m-Numb enhancement were resistant to H_2_O_2_-induced oxidative stress. Mt2 is important for the protection and regeneration of gastric lesions [40], [41], indicating that the process of gastric regeneration mediated by m-Numb is related to the anti-oxidant activity of Mt2, in addition to the enhancement of progenitor cell function due to the PSCA expression (Figure 7).

We used the acute gastric injury model with ethanol administration. In this model, the basal lamina is intact, and restitution can resurface the epithelium within 2–5 h [1], [2], [3], indicating that this is a suitable model to estimate the initial processes of gastric regeneration. In this study, we also observed severe gastric erosion 1 h after ethanol administration in both wild-type and Msi1-KO mice. Thus, Msi1 is a key regulatory factor for the initial regeneration of gastric mucosa. Furthermore, we need to investigate the whole regeneration process in Msi1-KO mice by using other models like the acetic acid model.

In conclusion, our findings, for the first time, provide direct evidence linking Msi1 to gastric regeneration using Msi1-KO mice. Furthermore, our results suggest that Msi1 post-transcriptionally enhances PTB domain-containing m-Numb protein expression after acute gastric mucosal injury. The enhancing m-Numb expression may be a novel and specific target for the gastric ulcer treatment and epithelial regeneration.

## Materials and Methods

### Ethics Statement

All experiments and procedures in our study were conducted with the approval of the Keio University Animal Research Committee (No. 08079). All the animal experiments were conducted according to the Guidelines for the Care and Use of Laboratory Animals of the Keio University School of Medicine, and every effort was made to minimize suffering of the animals. Mice were specific pathogen-free, and fed with complete pelleted chow and tap water *ad libitum* in a room with controlled light (12 h light, 12 h darkness).

### Animals

For generating the acute gastric mucosal injury model, ICR/CD1 background male Musashi-1-null mice [42] (10 to 20 weeks old) were used, with age-matched male wild-type ICR/CD1 littermate mice as controls. The mice were anesthetized with diethyl ether, and sacrificed after food deprivation for 18 h, after which the gastric tissues were dissected.

### Rapid Gastric Mucosal Injury

Absolute ethanol (8 ml/kg) was administered to the mice by gavage after 18 h of food deprivation. The stomachs were resected 1, 3, or 5 h after ethanol administration and opened along the greater curvature.

### Immunohistochemical Staining

The antibodies used for the immunohistochemical staining of the gastric mucosal paraffin sections of the mice were as follows: mouse monoclonal anti-H^+^, K^+^-ATPase alpha-subunit antibody (clone 1H9, 1∶300, Research Diagnostics Inc., Flanders, NJ, USA) against parietal cells, rabbit anti-mouse pepsinogen antibody (mPep) against zymogenic cells, and mouse monoclonal anti-Muc6 antibody (HIK1083, 1∶30, Kanto Chemical, Tokyo, Japan), originally raised against rat gastric mucin, to detect mucin-6 (Muc6) in the mammalian gastric fundic mucous neck cells and pyloric gland cells. Muc6 is identical to the class III mucin detected by paradoxical concanavalin A staining.

After opening the stomachs along the greater curvature, the specimens were fixed overnight in 10% neutralized buffered formalin and processed by the routine method for paraffin embedding. The sections were then stained with hematoxylin and eosin (H&E) using the standard technique.

For labeling with anti-mouse pepsinogen antibody, the sections were incubated in a citrate buffer solution (10 mM, pH 6.0) at 121°C for 15 min. For labeling with anti-H^+^, K^+^-ATPase, and anti-Muc6 antibodies, the sections were digested with proteinase K solution (Dako, Tokyo, Japan) for 4 min at room temperature. After antigen retrieval, endogenous peroxidase activity was quenched by treatment with 0.3% hydrogen peroxide in methanol for 15 min, and the sections were then treated with a blocking reagent (Protein Block Serum-Free, Dako), followed by incubation overnight with the primary antibodies at 4°C. Then, the sections were incubated with horseradish peroxidase (HRP)-labeled anti-rabbit IgG or HRP-labeled anti-mouse IgG (Histofine, Simple stain MAX-PO goat/rabbit/mouse; Nichirei, Tokyo, Japan) for 30 min at room temperature. This was followed by color development with a solution of 3, 3′-diaminobenzidine tetrahydrochloride. For single immunohistochemical staining, counterstaining was performed with Gill’s hematoxylin. The stained sections were examined using a light microscope equipped with a 3CCD digital camera (C7780; Hamamatsu Photonics, Hamamatsu, Japan).

### Evaluation of Gastric Epithelial Cell Degeneration

The area of gastric erosion in the gastric fundus, identified by positive staining of the parietal cells with the anti-H^+^, K^+^-ATPase alpha-subunit antibody, was estimated in sections stained with H&E. The area of gastric epithelial cell degeneration was expressed as a percentage of the total area.

### Reverse Transcription-polymerase Chain Reaction

Total RNA was isolated from the stomachs of mice, using the RNeasy Mini Kit (Qiagen, Germantown, MD, USA). First-strand cDNA synthesis was performed using a PrimeScript™ RT Reagent Kit (Takara Bio, Shiga, Japan).

Real-time PCR amplification was performed using a SYBR Premix Ex Taq Perfect Real Time kit (Takara Bio), in a Thermal Cycler Dice Real Time System (Takara Bio). The specific primers for amplifying mouse total *m-numb*, as well as the individual isoforms of *m-numb*, *LGR5*, *DCLK1*, *PSCA, Mt2, and glyceraldehyde-3-phosphate (GAPDH)* mRNAs (the latter as internal control) are listed in Table 1. The primers for the total *m-numb* mRNA amplified all the *m-numb* splicing variants. The 2-step PCR program was as follows: 95°C for 2 min followed by 40 cycles, each consisting of 95°C for 5 s and 60°C for 30 s.

**Table 1:** Primer sequences for quantitative RT-PCR.

Target gene	Forward primer (5'- 3')	Reverse primer (5'- 3')
total *m-numb*	ACTACGGCAAAGCTTCAGGA	ACGTGGCCGAGGTACTTAAC
*m-numb* PTB domain containing form (PTBL)	GAAAGGAAGTTCTTCAAAGG	CCACAACTCTGAGCCCATC
*m-numb* PTB domain-non-containing form (PTBS)	GATTGAAAGCTACGGGAAAG	AAGTTCCTATCTGGGGCACA
LGR5	CCAAGGGAGCGTTCACGGGC	CACGTAGCTGATGTGGTTGG
DCLK-1	CAGCCTGGACCAGCTGGTGG	TGACCAGTTGGGGTTCACAT
PSCA	CCGTCTTCTTTCTCCTGCTG	CGCGATGTAAAGCAACTGTG
Mt2	TCCACTCGCCATGGACCCCA	CAGCCCTGGGAGCACTTCGC
GAPDH	TGTGTCCGTCGTGGATCTGA	TTGCTGTTGAAGTCGCAGGAG

For semi-quantitative PCR, each cDNA was amplified using KOD plus Taq (Toyobo, Osaka, Japan). The primers used to detect the Numb1 and Numb2 mRNAs were PTBL forward, PRRL reverse (5′-ATGGCTGCAATTTCCTTGTT-3′), and PRRS reverse (5′-ACCCCACTCAGTCCCTTGTA-3′). The primers for GAPDH were the same as those used for the real-time PCR. The annealing temperature was 57°C for GAPDH and 60°C for Numb1 and Numb2.

### Western Blotting

The primary antibodies used for western blotting were as follows: goat polyclonal anti-m-Numb (1∶2000, Abcam, Cambridge, UK), which recognized the 14 C-terminal residues of m-Numb, rabbit polyclonal anti-p21 (1∶200, Santa Cruz Biotechnology, Santa Cruz, CA, USA), rat monoclonal anti-Msi1 clone 14H1 [43] (1∶1,000), monoclonal anti-Msi2 (1∶1,000, GeneTex, Irvine, CA, USA), and mouse monoclonal anti-β-actin clone AC-15 (1∶10,000, Sigma-Aldrich, St. Louis, MO, USA), which recognized β-actin used as the internal standard.

Each tissue was lysed in ice-cold RIPA lysis buffer containing a protease inhibitor cocktail (Sigma-Aldrich). The resulting protein lysate was boiled in LDS sample buffer (Invitrogen) and separated by 4–12% NuPAGE Bis-Tris gel (Invitrogen, Carlsbad, CA, USA) electrophoresis using MOPS running buffer (Invitrogen). After electrophoresis, gels were blotted onto polyvinylidene difluoride membranes in NuPAGE Transfer buffer (Invitrogen). Membranes were blocked with a solution containing BlockAce (Dainippon Sumitomo Pharma Co., Osaka, Japan) for 1 h and incubated overnight with the primary antibody at 4°C, followed by incubation with HRP-conjugated secondary antibody for 1 h at room temperature. Thereafter, a chemiluminescence solution (GE Healthcare, Buckinghamshire, UK) was applied, and images were acquired using a FUJI LAS4000mini (GE Healthcare).

### Polysome Gradient Fractionation From Gastric Tissues

Preparation of gastric tissue for polysome analysis was performed essentially according to previously described methods [44]. A 20-mg piece of solid tissue was lysed in 1 ml of lysis buffer (10 mM Tris-HCl at pH 8.0, 150 mM NaCl, 5 mM MgCl_2_, 1% Nonidet-P40, 40 mM dithiothreitol, 1000 U/ml RNAase inhibitor (Toyobo), 40 mM vanadyl ribosyl complex (New England Bio Labs, Ipswich, MA, USA) supplemented with 1% deoxycholic acid sodium salt monohydrate (Nacalai Tesque, Inc., Kyoto, Japan). After the cells were lysed by pipetting, the nuclei were removed by centrifugation (12,000 × *g* for 10 s at 4°C). The supernatant was supplemented with 500 µL of extraction buffer (0.2 M Tris-HCl at pH 7.5, 0.3 M NaCl), 150 µg/ml cycloheximide, 650 µg/ml heparin, and 10 mM phenylmethylsulfonyl fluoride, and then centrifuged (12,000 × *g* for 5 min at 4°C) to remove mitochondria and membranous debris.

The supernatant was then layered onto a 36-ml linear sucrose gradient (15–40% sucrose [w/v], supplemented with 10 mM Tris-HCl at pH 7.5, 140 mM NaCl, 1.5 mM MgCl_2_, 10 mM dithiothreitol, 100 µg/ml cycloheximide, 0.5 mg/ml heparin) and centrifuged in a Beckman SW28 rotor for 205 min at 28,000 rpm at 4°C, with the brake off. Fractions (2 ml) were collected by adding 40% ethanol and 0.6 M guanidine (final concentration). Then, 100 µg of proteinase K in 1% SDS and 10 mM EDTA were added, and digestion was allowed to continue for 30 min at 37°C.

Total RNA from each fraction was recovered by extraction with an equal volume of phenol-chloroform-isoamyl alcohol, followed by ethanol precipitation. The quantity of *m-numb mRNA* in each fraction was determined using the total m-Numb primers and One-Step SYBR® PrimeScript™ PLUS RT-PCR kit (Takara Bio). These data are presented as percentage of the total amount of RNA in each fraction.

### Preparation of Lentivirus Particles

Lentiviral mouse Numb1 and Numb2 expression vectors were constructed by inserting the corresponding cDNA sequence into the CSII-CMV-MCS-IRES2-Bsd lentiviral expression vector (kindly provided by H. Miyoshi, RIKEN). A shRNA-containing pGIPZ lentiviral vector was acquired from Open Biosystems (Huntsville, AL, USA). The respective lentiviral vectors were co-transfected with lentiviral packaging vectors into HEK-293T cells to produce mouse Numb1, Numb2, and shRNA-carrying lentiviral particles. Culture supernatants were collected 48 h after incubation and filtered through 0.45-µM membranes to generate cell-free virus.

### Knockdown Expression using shRNA-containing Lentivirus Particles

Human gastric cell line N87 cells were obtained from American Type Culture Collection. The cells were transduced by the shRNA-carrying lentiviral particles using Viromag R/L beads (OZbiosciences, Marseille, France) at multiplicities of infection 100, and these cells were cultured for a week in the presence of puromycin (10 µg/ml). A non-silencing shRNA control with no homology to known mammalian genes was used as negative control for the knockdown experiment.

### Over Expression of m-numb

An overexpression study of m-Numb was carried out in the mouse gastric cell line MGE507, which was established from transgenic mice harboring temperature-sensitive simian virus 40 large T antigen [45]. The cells expressed mRNAs of muc5ac, the α-subunit of the H^+^, K^+^-ATPase, and pepsinogen F [45]. The cells were transduced by the mouse Numb1 and Numb2-carrying lentiviral particles using Viromag R/L beads at multiplicities of infection 100, and these cells were cultured for a week in the presence of blasticidin (5 µg/ml). A LacZ gene- carrying lentiviral particle was used as a control.

### Cell Viability Assay

To evaluate the rate of cell proliferation, MGE507 cells were plated at a density of 5×10^3^ cells/well on 96-well plate in Dulbecco’s modified Eagle medium/Ham’s F-12 (DMEM/F-12: Sigma-Aldrich) supplemented with 1% FBS. After 24 hour’s incubation at 37°C, Cell Count Reagent SF (Nacalai Tesque) were added to the culture medium at 10% of final concentration and further incubated for 3 h at the end of the culture. The absorbance at 450 nm (reference at 650 nm) of each well was measured. In the experiments in which the cytotoxic effect of H_2_O_2_ was analyzed, MGE507 cells were plated at a density of 10^4^ cells/well on 96-well plate in DMEM/F-12 medium supplemented with 5% FBS. After 18 h at 37°C, the cells were washed, incubated for 1 h in serum free medium. Then, they were exposed to 500 µM H_2_O_2_ in serum free medium and incubated for another 2 h. Finally, they were washed twice with serum free medium, 10 µl of Cell Count Reagent SF were added to 100 µl of the culture for 3 h at the end of the culture. The absorbance at 450 nm (reference at 650 nm) of each well was measured.

### Statistical Analysis

All the data were expressed as mean (SD). To define statistically significant differences between the 2 groups, the data were subjected to Student’s *t*-test or Welch’s *t*-test. The analysis was performed using a personal computer with the DA Stats software (ver. 1.0, freeware soft, copyright® 1993, by Dr. O. Nagata), after examination of the variances of the data using the F-test.

For multiple-group comparisons, comparisons between groups were performed using one-way ANOVA, followed by multiple comparison testing using Tukey’s test, with PRISM4 software for Macintosh (GraphPad Software Inc., San Diego, CA, USA).

## Supporting Information

Figure S1 **Schematic representation of** ***m-Numb*** **splicing variant.**
(PDF)

Figure S2 **Immunohistochemical analysis in the control group.** Wild-type (A, C, and E) and Msi1-KO (B, D, and F) mice were administered water. Sections of the gastric mucosa from each mouse were then stained using anti-H^+^, K^+^-ATPase- (A and B), anti-Muc6- (C and D), and anti-pepsinogen- (E and F) antibodies. Bar = 100 µm.
(PDF)

Figure S3 **m-Numb expression in the mouse gastric tissue.** The stomachs of sham-treated wild-type mice were fixed in 4% paraformaldehyde, and frozen sections were prepared. Each of the sections was stained using anti-m-Numb primary antibody and Alexa-488-conjugated anti-rabbit IgG secondary antibody. Counterstaining was performed using the anti-H^+^, K^+^-ATPase alpha-subunit primary antibody and Alexa-568-conjugated anti-mouse IgG secondary antibody. Bars = 100 µm.
(PDF)

Figure S4 **Expression of m-Numb proteinin in the various tissues of the wild-type and Msi1-KO mice.** The amount of protein from the tissues loaded in each lane for western blotting was as follows; cerebrum, cerebellum and lung; 5 µg/lane, others; 30 µg/lane. Wt; w ild-type, KO; Msi1-KO.
(PDF)

Figure S5 **Numb PTBS RT minus quantitative PCR assay.** Amplification of Numb-PTBS variant mRNA was performed by realtime quantitative PCR using templates of RT plus or minus RNAs from sham-treated wild-type and Msi1-KO mice of stomach. cDNA; RT plus templates, RT(-); RT minus templates.
(PDF)

Figure S6 **Variation of m-Numb 5′-UTR.** (A) Schematic representation of human Numb1 reference sequence (accession NM_001005743.1) (B) Ratio of each *m-Numb* 5′-UTR variant. *E. coli* transformed with the ligation product of the TA-cloning vector and RACE PCR amplicon of *m-Numb* was cultured, and the type of 5′-UTR variant in the resulting colony determined by DNA sequencing and PCR. Fifty colonies each resulting from stomach and brain constructs were sequenced.
(PDF)

Methods S1 **Fluorescent immunostaining of the m-Numb protein in the mouse gastric tissues.** The mouse gastric tissue specimens were fixed with 4% paraformaldehyde and frozen sections were prepared. For fluorescent immunostaining of the human gastric tissues, paraffin sections of human gastric tissue specimens were deparaffinized, rehydrated and treated with antigen retrieval solution at 90°C for 20 min (Nacalai tesque, Kyoto, Japan). The sections were then treated with a blocking reagent and incubated overnight with rabbit anti-m-Numb antibody (1∶200, Abcam, Cambridge, UK) at 4°C. The sections from the mice were further incubated with mouse monoclonal anti- H^+^, K^+^-ATPase antibody, followed by washing with PBS and then incubation with Alexa-488-labeled anti-rabbit IgG and Alexa-568-labeled anti-mouse IgG (Molecular Probes, Eugene, OR) for 2 h at room temperature. In the human gastric tissue sections, nuclei were visualized with 4', 6-diamidino-2-phenylindole (DAPI) (Sigma-Aldrich). The prepared sections were examined under a Zeiss LSM510 laser scanning confocal microscope (Zeiss Microimaging, Thornwood, NY). ***RACE analysis:*** Rapid amplification of cDNA 3′-ends (3′-RACE) and 5′-ends (5′-RACE) experiments were performed using SMARTer™ RACE cDNA Amplification Kit (Clontech, Palo Alto, CA, USA). Human normal brain and stomach RNA (Takara Bio) were used as templates. The cDNA generated by RACE was amplified by PCR using the universal primer A mix, provided by the manufacturer, and a gene-specific primer. The gene-specific primers for 3′-RACE and 5′-RACE were as follows: 3′-RACE: 5′-CAGCAGACAGGCATACAGAGGTTCCT-3′, and 5′-RACE: 5′-TCCGGTGCGAACGCCTTCTT-3′. The resulting PCR amplicon was ligated into the pMD20 TA cloning vector (Takara Bio). The ligation products were then used to transform *E. coli* DH5α competent cells. After transformation, the integrity of the inserted sequence was determined by DNA sequencing.
(DOC)

Result S1 **UTR analysis.** Only full-length 3′-UTR sequence of *m-Numb mRNA* was obtained by 3′-RACE in both stomach and brain; this sequence corresponded to the reference sequence (positions 2276–3644 in accession NM_001005743.1; Figure S6A). On the other hand, 5′-RACE analysis revealed splicing variants lacking exon 2 (ΔEx2) or exon 3 (ΔEx3) in the 5′-UTR sequence of *m-Numb mRNA*. Among 50 colonies of transformed *E. coli* derived from stomach or brain products of 5′-RACE, the ΔEx2 variant of the 5′-UTR was only detected in the colonies derived from the stomach (Figure S6B).
(DOC)
