# Supplementary figures and images for: Musashi-1 Post-Transcriptionally Enhances Phosphotyrosine-Binding Domain-Containing m-Numb Protein Expression in Regenerating Gastric Mucosa
Source: PLoS One. 2013 Jan 4;8(1):e53540. doi: 10.1371/journal.pone.0053540 (PMC3537613; doi:10.1371/journal.pone.0053540)

### Figure S1

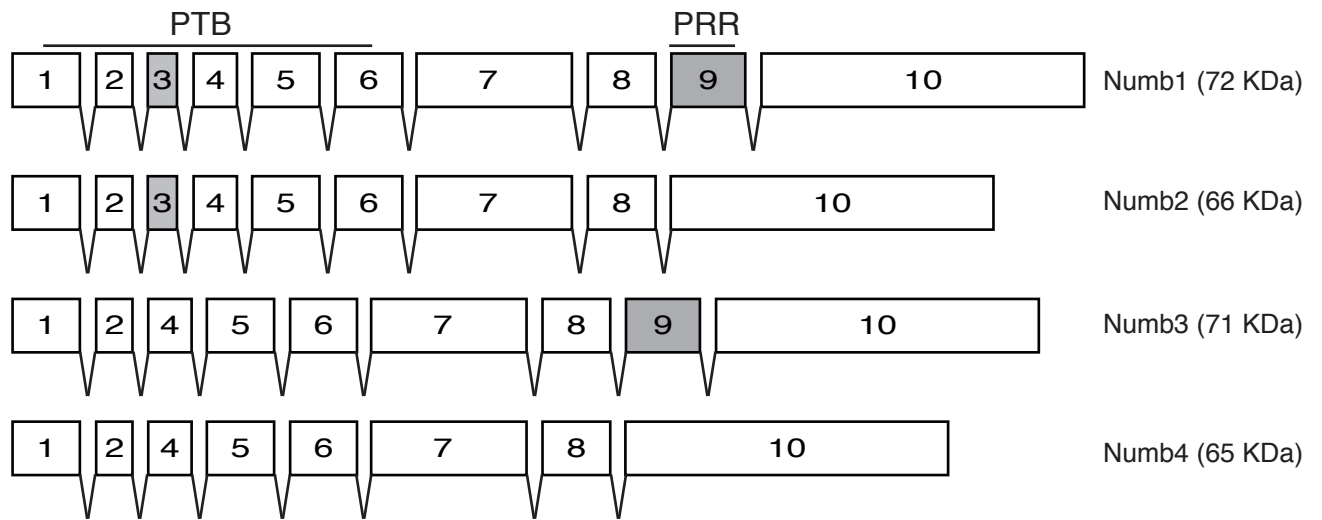

Supplement: Figure S1 — Schematic representation of m-Numb splicing variant. (PDF) [file pone.0053540.s001.pdf]

Figure S2

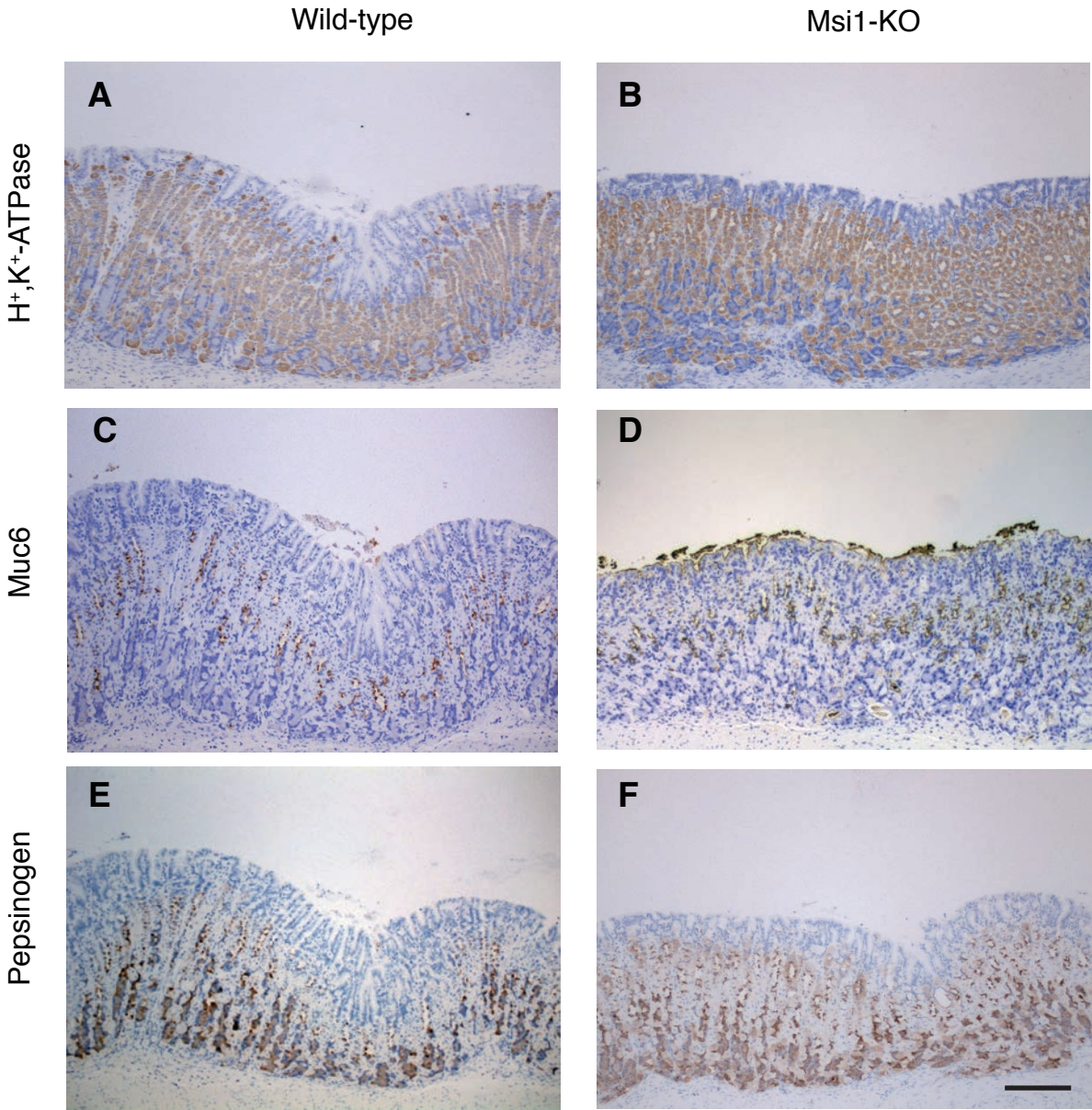

Supplement: Figure S2 — Immunohistochemical analysis in the control group. Wild-type (A, C, and E) and Msi1-KO (B, D, and F) mice were administered water. Sections of the gastric mucosa from each mouse were then stained using anti-H+, K+-ATPase- (A and B), anti-Muc6- (C and D), and anti-pepsinogen- (E and F) antibodies. Bar = 100 µm. (PDF) [file pone.0053540.s002.pdf]

**Figure S3**

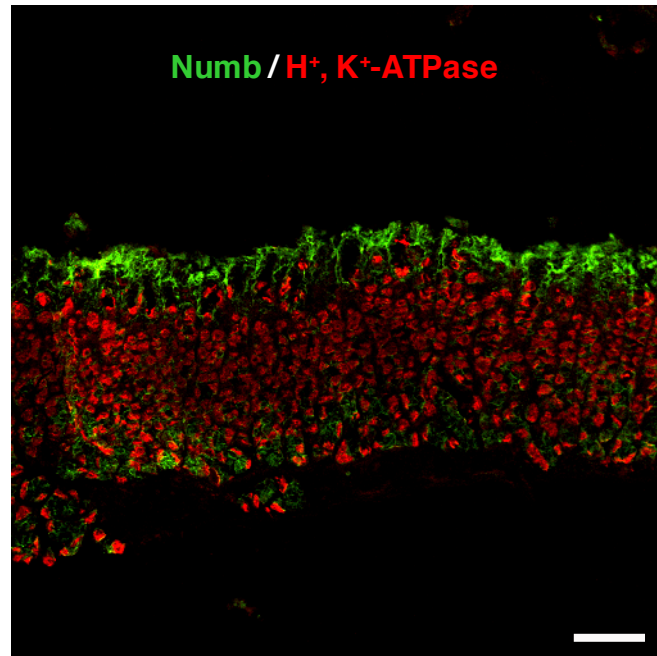

Supplement: Figure S3 — m-Numb expression in the mouse gastric tissue. The stomachs of sham-treated wild-type mice were fixed in 4% paraformaldehyde, and frozen sections were prepared. Each of the sections was stained using anti-m-Numb primary antibody and Alexa-488-conjugated anti-rabbit IgG secondary antibody. Counterstaining was performed using the anti-H+, K+-ATPase alpha-subunit primary antibody and Alexa-568-conjugated anti-mouse IgG secondary antibody. Bars = 100 µm. (PDF) [file pone.0053540.s003.pdf]

Figure S4

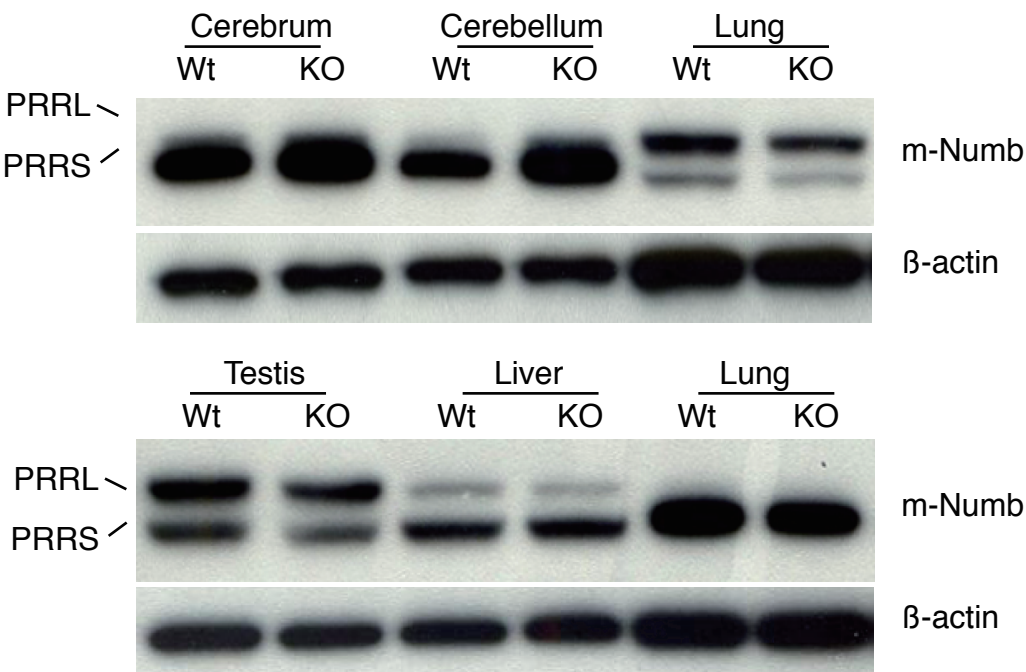

Supplement: Figure S4 — Expression of m-Numb proteinin in the various tissues of the wild-type and Msi1-KO mice. The amount of protein from the tissues loaded in each lane for western blotting was as follows; cerebrum, cerebellum and lung; 5 µg/lane, others; 30 µg/lane. Wt; w ild-type, KO; Msi1-KO. (PDF) [file pone.0053540.s004.pdf]

**Figure S5.**

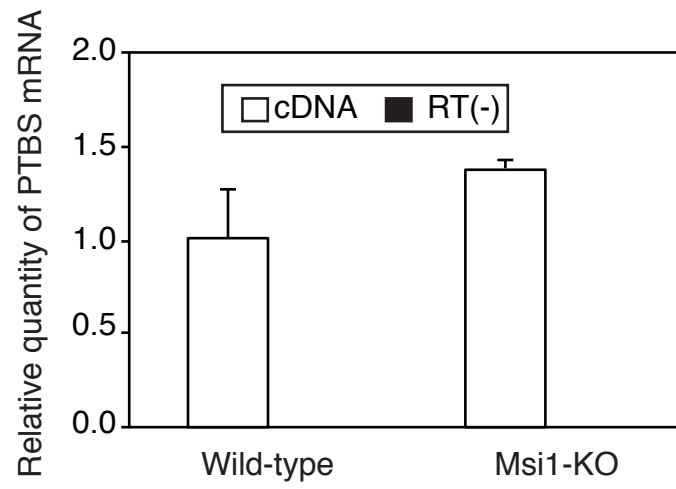

Supplement: Figure S5 — Numb PTBS RT minus quantitative PCR assay. Amplification of Numb-PTBS variant mRNA was performed by realtime quantitative PCR using templates of RT plus or minus RNAs from sham-treated wild-type and Msi1-KO mice of stomach. cDNA; RT plus templates, RT(-); RT minus templates. (PDF) [file pone.0053540.s005.pdf]

Figure S6

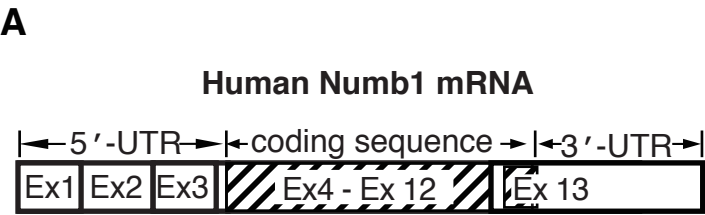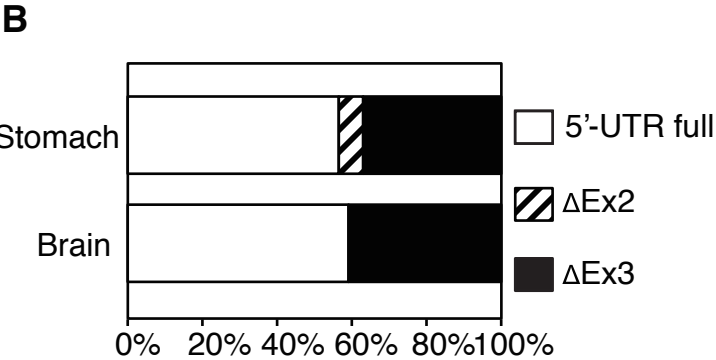

Supplement: Figure S6 — Variation of m-Numb 5′-UTR. (A) Schematic representation of human Numb1 reference sequence (accession NM_001005743.1) (B) Ratio of each m-Numb 5′-UTR variant. E. coli transformed with the ligation product of the TA-cloning vector and RACE PCR amplicon of m-Numb was cultured, and the type of 5′-UTR variant in the resulting colony determined by DNA sequencing and PCR. Fifty colonies each resulting from stomach and brain constructs were sequenced. (PDF) [file pone.0053540.s006.pdf]
