# Supplementary material for: Musashi-1 Post-Transcriptionally Enhances Phosphotyrosine-Binding Domain-Containing m-Numb Protein Expression in Regenerating Gastric Mucosa
Source: PLoS One. 2013 Jan 4;8(1):e53540. doi: 10.1371/journal.pone.0053540 (PMC3537613; doi:10.1371/journal.pone.0053540)
Supplement: Methods S1 — Fluorescent immunostaining of the m-Numb protein in the mouse gastric tissues. The mouse gastric tissue specimens were fixed with 4% paraformaldehyde and frozen sections were prepared. For fluorescent immunostaining of the human gastric tissues, paraffin sections of human gastric tissue specimens were deparaffinized, rehydrated and treated with antigen retrieval solution at 90°C for 20 min (Nacalai tesque, Kyoto, Japan). The sections were then treated with a blocking reagent and incubated overnight with rabbit anti-m-Numb antibody (1∶200, Abcam, Cambridge, UK) at 4°C. The sections from the mice were further incubated with mouse monoclonal anti- H+, K+-ATPase antibody, followed by washing with PBS and then incubation with Alexa-488-labeled anti-rabbit IgG and Alexa-568-labeled anti-mouse IgG (Molecular Probes, Eugene, OR) for 2 h at room temperature. In the human gastric tissue sections, nuclei were visualized with 4', 6-diamidino-2-phenylindole (DAPI) (Sigma-Aldrich). The prepared sections were examined under a Zeiss LSM510 laser scanning confocal microscope (Zeiss Microimaging, Thornwood, NY). RACE analysis: Rapid amplification of cDNA 3′-ends (3′-RACE) and 5′-ends (5′-RACE) experiments were performed using SMARTer™ RACE cDNA Amplification Kit (Clontech, Palo Alto, CA, USA). Human normal brain and stomach RNA (Takara Bio) were used as templates. The cDNA generated by RACE was amplified by PCR using the universal primer A mix, provided by the manufacturer, and a gene-specific primer. The gene-specific primers for 3′-RACE and 5′-RACE were as follows: 3′-RACE: 5′-CAGCAGACAGGCATACAGAGGTTCCT-3′, and 5′-RACE: 5′-TCCGGTGCGAACGCCTTCTT-3′. The resulting PCR amplicon was ligated into the pMD20 TA cloning vector (Takara Bio). The ligation products were then used to transform E. coli DH5α competent cells. After transformation, the integrity of the inserted sequence was determined by DNA sequencing. (DOC) [file pone.0053540.s007.doc]

**Supplementary materials and methods**

**Methods**

**Fluorescent immunostaining of the m-Numb protein in the mouse gastric tissues**

The mouse gastric tissue specimens were fixed with 4% paraformaldehyde and frozen sections were prepared. For fluorescent immunostaining of the human gastric tissues, paraffin sections of human gastric tissue specimens were deparaffinized, rehydrated and treated with antigen retrieval solution at 90°C for 20 min (Nacalai tesque, Kyoto, Japan). The sections were then treated with a blocking reagent and incubated overnight with rabbit anti-m-Numb antibody (1:200, Abcam, Cambridge, UK) at 4°C. The sections from the mice were further incubated with mouse monoclonal anti- H^+^, K^+^-ATPase antibody, followed by washing with PBS and then incubation with Alexa-488-labeled anti-rabbit IgG and Alexa-568-labeled anti-mouse IgG (Molecular Probes, Eugene, OR) for 2 h at room temperature. In the human gastric tissue sections, nuclei were visualized with 4', 6-diamidino-2-phenylindole (DAPI) (Sigma-Aldrich). The prepared sections were examined under a Zeiss LSM510 laser scanning confocal microscope (Zeiss Microimaging, Thornwood, NY).

***RACE analysis***

Rapid amplification of cDNA 3′-ends (3′-RACE) and 5′-ends (5′-RACE) experiments were performed using SMARTer^TM^ RACE cDNA Amplification Kit (Clontech, Palo Alto, CA, USA). Human normal brain and stomach RNA (Takara Bio) were used as templates. The cDNA generated by RACE was amplified by PCR using the universal primer A mix, provided by the manufacturer, and a gene-specific primer. The gene-specific primers for 3′-RACE and 5′-RACE were as follows: 3′-RACE: 5′-CAGCAGACAGGCATACAGAGGTTCCT-3′, and 5′-RACE: 5′-TCCGGTGCGAACGCCTTCTT-3′. The resulting PCR amplicon was ligated into the pMD20 TA cloning vector (Takara Bio). The ligation products were then used to transform *E. coli* DH5α competent cells. After transformation, the integrity of the inserted sequence was determined by DNA sequencing.
