# Supplementary material for: Musashi-1 Post-Transcriptionally Enhances Phosphotyrosine-Binding Domain-Containing m-Numb Protein Expression in Regenerating Gastric Mucosa
Source: PLoS One. 2013 Jan 4;8(1):e53540. doi: 10.1371/journal.pone.0053540 (PMC3537613; doi:10.1371/journal.pone.0053540)
Supplement: Result S1 — UTR analysis. Only full-length 3′-UTR sequence of m-Numb mRNA was obtained by 3′-RACE in both stomach and brain; this sequence corresponded to the reference sequence (positions 2276–3644 in accession NM_001005743.1; Figure S6A). On the other hand, 5′-RACE analysis revealed splicing variants lacking exon 2 (ΔEx2) or exon 3 (ΔEx3) in the 5′-UTR sequence of m-Numb mRNA. Among 50 colonies of transformed E. coli derived from stomach or brain products of 5′-RACE, the ΔEx2 variant of the 5′-UTR was only detected in the colonies derived from the stomach (Figure S6B). (DOC) [file pone.0053540.s008.doc]

**Results**

***UTR analysis***

Only full-length 3′-UTR sequence of *m-Numb mRNA* was obtained by 3′-RACE in both stomach and brain; this sequence corresponded to the reference sequence (positions 2276–3644 in accession NM_001005743.1; Figure S6A). On the other hand, 5′-RACE analysis revealed splicing variants lacking exon 2 (∆Ex2) or exon 3 (∆Ex3) in the 5′-UTR sequence of *m-Numb mRNA*. Among 50 colonies of transformed *E. coli* derived from stomach or brain products of 5′-RACE, the ∆Ex2 variant of the 5′-UTR was only detected in the colonies derived from the stomach (Figure S6B).
